# Gamma-Irradiation Effects on the Spectral and Amplified Spontaneous Emission (ASE) Properties of Conjugated Polymers in Solution

**DOI:** 10.3390/polym9010007

**Published:** 2016-12-28

**Authors:** Mohamad S. AlSalhi, Saradh Prasad, D. Devaraj, Ziad S. Abo Mustafa

**Affiliations:** 1 Department of Physics and Astronomy, College of Science, King Saud University, Riyadh 11451, Saudi Arabia; srajendra@ksu.edu.sa (S.P.); ziadabomustafa@yahoo.com (Z.S.A.M.); 2 Research Chair on Laser Diagnosis of Cancers, College of Science, King Saud University, Riyadh 11451, Saudi Arabia; 3 Department of Electrical and Electronics, College of Engineering, Kalasalingam University, Anad Nagar, Krishnankoil, Virudhunagar 626190, Tamil Nadu, India; ddevaraj.klu@gmail.com

**Keywords:** conjugated *co*-polymer BEHP-*co*-MEH–PPV, MEH–PPV, gamma-irradiation effect, amplified spontaneous emission profile

## Abstract

In this paper, we investigate the effects of gamma (γ) radiation on the spectral and mplified spontaneous emission (ASE) properties of two conjugated polymers (CPs) viz., poly [2-methoxy-5-(2-ethylhexyloxy)-1,4-phenylenevinylene] (MEH–PPV) (CPM) and poly{[2-[2′,5′-*bis*(2″-ethylhexyloxy)phenyl]-1,4-phenylenevinylene]-*co*-[2-methoxy-5-(2′-ethylhexyloxy)-1,4-phenylene vinylene]} (BEHP-*co*-MEH–PPV) (BMP) in tetrahydrofuran (THF). Gamma irradiation strongly affected the photophysical properties of these CPs. To explore these changes, gamma radiation, in the range of 2–50 kGy, was used to maintain the temperature at 5 °C constant for all doses at a dose rate of 12.67 kGy/h, using a ^60^Co gamma ray. The ASE profiles of the CPs in THF were obtained under the high power excitation of a Nd:YAG laser (355 nm), pre- and post-radiation. The result revealed a dramatic blue shift of the fluorescence and the ASE spectra after gamma irradiation. This shift in the luminescence and ASE spectra could be a response to the conformational disorders such as gamma irradiation-induced polymer crosslinking, which was verified using Raman spectra, FTIR, and swelling experiments. This could be the first report on the effect of gamma radiation on the ASE properties of conjugated polymers.

## 1. Introduction

Light emitting diodes (LEDs) based on conjugated polymers have generated great interest because of their potential applicability to large areas and flat panel displays, their ability to operate at relatively low voltage, their low cost, and their easy fabrication [1]. Among conjugated polymers, the polyphenylene-vinylene (PPV) family has drawn considerable attention for its significant optical and electrical properties [2,3,4,5,6,7]. Conjugated polymers have also been investigated as potential radiation detectors [8,9,10,11].

Conjugated polymers can be excited via charge injection or photo-excitation. These macromolecules show spectral properties similar to those of optically active small organic molecules, such as laser dyes. Conjugated polymers possess characteristics of broadband emission appropriate for tunable lasers, with strong powers of absorption and emission, a large Stokes shift, high luminescence, and substantial quantum yield. Many fluorescent organic molecules such as laser dyes reveal a very low quantum yield at higher concentrations because of reabsorption and are unable to emit laser light, especially in a solid film configuration. In contrast, conjugated polymers can emit fluorescence and laser light as neat solid films, even at higher concentrations [12].

In 1992, Moses produced a laser action using a liquid state conjugated polymer (MEH–PPV), operating in the portion of the yellow/red wavelength. The MEH–PPV performed just as efficiently as the conventional laser dye rhodamine 6G [13].

Recently, a study was conducted on the gamma-irradiation effects on the spectral properties of poly[2-methoxy-5-(2-ethylhexyloxy)-p-phenylenevinylene] (MEH–PPV) dissolved in tetrahydrofuran (THF). The experimental results confirmed the high sensitivity of the MEH–PPV solution to low-dose irradiation at low concentrations. The radiation-induced ionization caused the dissociation of the C–H bonds of the polymer, which may have caused the crosslinking of the polymer chains while generating hydrogen gas [14,15]. Ion irradiation has been recognized as a promoter of hydrogen loss in organic materials [16,17], and ionization is mainly accountable for the chain scission and/or crosslinking in polymers [14]. In a closely related study, the effects of low-dose gamma radiation on the optical properties of MEH–PPV in solution and thin films were explored [18].

The high atomic number of bismuth iodide and the high-sensitivity photoluminescence quenching of the conjugated polymers are leveraged in their nanocomposites to detect high-energy photons [19]. The ionizing radiation effects (neutron and gamma) on conducting polyaniline were studied for various doses, from 6 to 504 Gy and 15 to 41.9 kGy [20].

The lasing characteristics of the BEHP-co-MEH–PPV (BMP) as a quasi-periodic photonic crystal (QPCs) produced by the nano-etching process were reported by G. Yang et al. [21]. They generated QPCs in the form of octagonal quasicrystal slabs with a single-defect microcavity at low-index contrast. These QPCs were produced under optical pumping using the third harmonic of a Nd:YAG laser (355 nm, 30 ps, 10 Hz) to produce ASE at 610 nm with a full width half-maximum (FWHM) of 1 nm, and this peak (610 nm) could be attributed to the excimer of the MEH–PPV segment of BMP [22]. Organic photovoltaic cells were fabricated by sandwiching a composite active organic electronic material layer of the conjugated co-polymer BMP mixed with ZnO nanoparticles, between two metallic conductors [23].

The amplified spontaneous emission (ASE) spectra of the conjugated copolymer BMP was accomplished in organic solutions under optical pumping by the third harmonic of the Nd:YAG (355 nm). From the results, it was evident that the BMP produced laser light at a 560 nm wavelength. This peak (560 nm) could be attributed to the monomeric state of the MEH–PPV as a segment [22].

In a prior paper released by us, the MEH–PPV fluorescence at low concentrations was observed in two bands of 560 and 600 nm, which are ascribed to the *monomer* and *excimer* states, respectively. Under appropriate concentrations and pump power energy, the MEH–PPV generated the ASE in its excimeric state (600 nm), although the BEHP-*co*-MEH–PPV could produce only one band at 560 nm (monomer) for similar concentrations [22]. The effects of the different gamma-irradiation doses on the spectral properties and amplified spontaneous emission of the MEH–PPV and BMP are reported in the current study. The results show that, for various gamma ray doses, the fluorescence spectra of these conjugated polymers revealed a blue shift, which were more pronounced at lower concentrations and higher doses. The principal difference was that the BMP produced the ASE at much higher concentrations than did the MEH–PPV. The BMP could also withstand higher gamma-radiation doses when compared with the MEH–PPV. Gamma irradiation of the CPs could find use as a new method of obtaining tunable optoelectronic devices and lasers. However, the photochemical stability of the gamma-exposed samples was considerably lower than that seen in the unexposed samples.

## 2. Materials and Methods

**Figure 1:**
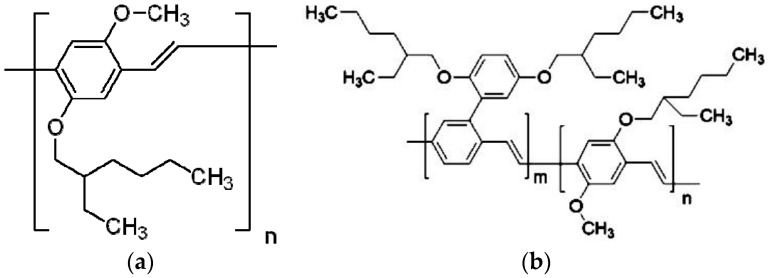
(**a**) Molecular structures of MEH–PPV; (**b**) molecular structures of BEHP-*co*-MEH–PPV (BMP).

The polymer poly [2-methoxy-5-(2-ethylhexyloxy)-1,4-phenylenevinylene] (MEH–PPV) and poly{[2-[2′,5′-*bis*(2″-ethylhexyloxy)phenyl]-1,4-phenylenevinylene]-*co*-[2-methoxy-5-(2′-ethylhexyloxy)-1,4-phenylenevinylene]} (BEHP-*co*-MEH–PPV or BMP), purchased from Sigma-Aldrich (St. Louis, MO, USA), were used as received. The sample purity, when tested via thin layer chromatography (TLC), was greater than 95%. The molecular weight *M*_w_ (GPC) was 100,000 and 130,000 (PS Standard) for MEH–PPV and BMP, respectively, molecular structures of both CPs are shown in Figure 1a,b. A 60:40 ratio was observed between the two segments of the polymer, BEHP–PPV and MEH–PPV. The BMP was dissolved in the tetrahydrofuran (THF) (spectroscopic grade of 99.8% purity) in a wide concentration range.

The absorption spectra were recorded using a Perkin Elmer lambda 950 spectrophotometer (Waltham, MA, USA) across the 100 to 1100 nm range, and the fluorescence spectra were recorded using a Perkin Elmer LS 55 spectrofluorometer (Waltham, MA, USA) with a scan range of 200 to 1000 mm at room temperature. The excitation wavelength was 355 nm [24].

**Figure 2:**
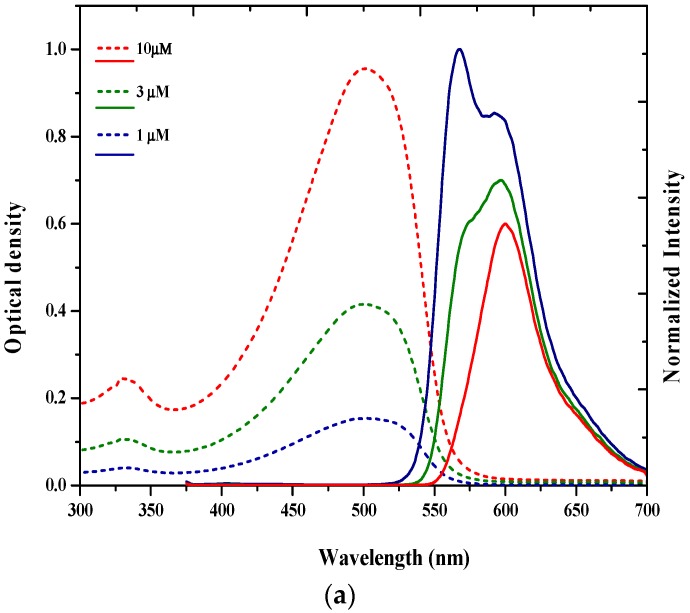

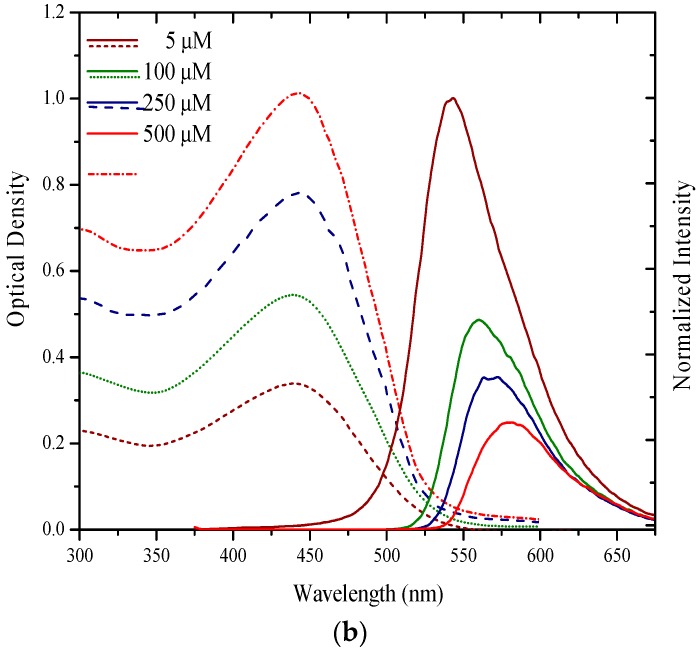
(**a**) Absorption and fluorescence spectra of MEH–PPV; (**b**) Absorption and fluorescence spectra of BMP. (In the above figures, discontinuous line represents absorption and continuous line represents fluorescence).

The excitation source was the third harmonic (355 nm) of an Nd:YAG laser at a 6 ns pulse width. A 5 cm focal length quartz cylindrical lens was used to focus the UV laser pulse. The focused pulse was transversely applied to excite the CPs solution. The cuvette was maintained at a tilt to avoid feedback (Figure 2). At a suitable pump power and CPs concentration, an ASE beam was achieved, which exited as a cone of light. The light thus emanated was collected by a 1 mm entrance slit of a spectrograph with a linear array charge-coupled device (CCD) [Ocean optics Spectroscopy, USB4000-XR1-ES, (Maybachstrasse, Ostfildern, Germany)] to record the ASE spectral features [25].

The gamma ray source used was ^60^Co, with radiation ranging from doses of 5 to 20 kGy, at 5 °C constant temperature for all the doses at a dose rate of 12.67 kGy/h.

The pre- and post-gamma-irradiated samples were spin coated on a quartz substrate up to a thickness of 1 μm. Then, the CPs were carefully peeled off from the quartz substrate. These samples were used to measure the FT-IR spectra. FT-IR spectroscopy was performed using an advanced attenuated total reflectance (ATR) measurement instrument (Perkin Elmer Spotlight 150i, Waltham, MA, USA) for a range of 4000 to 550 cm^−1^. Waters gel permeation chromatograph (GPC, Model 440, Waters Corporation, Milford, MA, USA) was used for gel permeation chromatography (GPC) measurements. The drop-casted samples were then cut into 6-mg-sized, rectangular thin films. They were then dropped into a beaker containing benzene for 10 min, removed, and immediately weighed. The erosion of the sample was found to be negligible, when compared with the weight gained. The swelling ratio can be calculated by applying the formula given below:

$$\mathrm{Swelling}\;\mathrm{ratio}=\frac{w_{s}-w_{d}}{w_{d}}$$

where *w_d_* is the dry weight of the polymer, and *w_s_* is the weight of the swollen polymer [26].

## 3. Results and Discussion

### 3.1. Spectral Properties

The absorption and fluorescence spectra of polymer poly[2-methoxy-5-(2-ethylhexyloxy)-1,4-phenylenevinylene] (MEH–PPV) in tetrahydrofuran (THF) at room temperature for different concentrations from 1 to 10 µM were recorded. Figure 2a shows the presence of two absorption bands (in a dashed line), one at 333 nm and another at 500 nm [19]. The shape of the absorption spectra remained unchanged regardless of the concentration, although the optical density rose with increasing concentrations of MEH–PPV.

The fluorescence spectra of the MEH–PPV in THF for different concentrations (1, 3, and 10 µM) were recorded. Figure 2a shows (in solid lines) two bands in the fluorescence spectrum of MEH–PPV viz., 560 and 600 nm. At a higher concentration of 10 μM, the fluorescence spectrum dramatically changed. The band at 560 nm was almost completely absent. These fluorescence spectral changes caused by the different concentrations could explain why MEH–PPV exists in the excimeric state, which is inter-chain interactive [27].

Similarly, the absorption and fluorescence spectra of the BEHP-*co*-MEH–PPV (BMP) in tetrahydrofuran (THF) were recorded pre-gamma irradiation. The solutions ranged in concentrations from 5 to 500 µM. The two peaks in the absorption spectra were observed at around 300 and 450 nm. The shapes of the absorption spectra remained the same irrespective of the concentration, although the optical density rose as the concentrations of BMP increased, as shown in Figure 2b. The spectra from 6 to 49 µM are noted to be absent in Figure 2b, because the change in optical density is low; the absorbance is only for comparison and not to scale [22].

**Figure 3:**
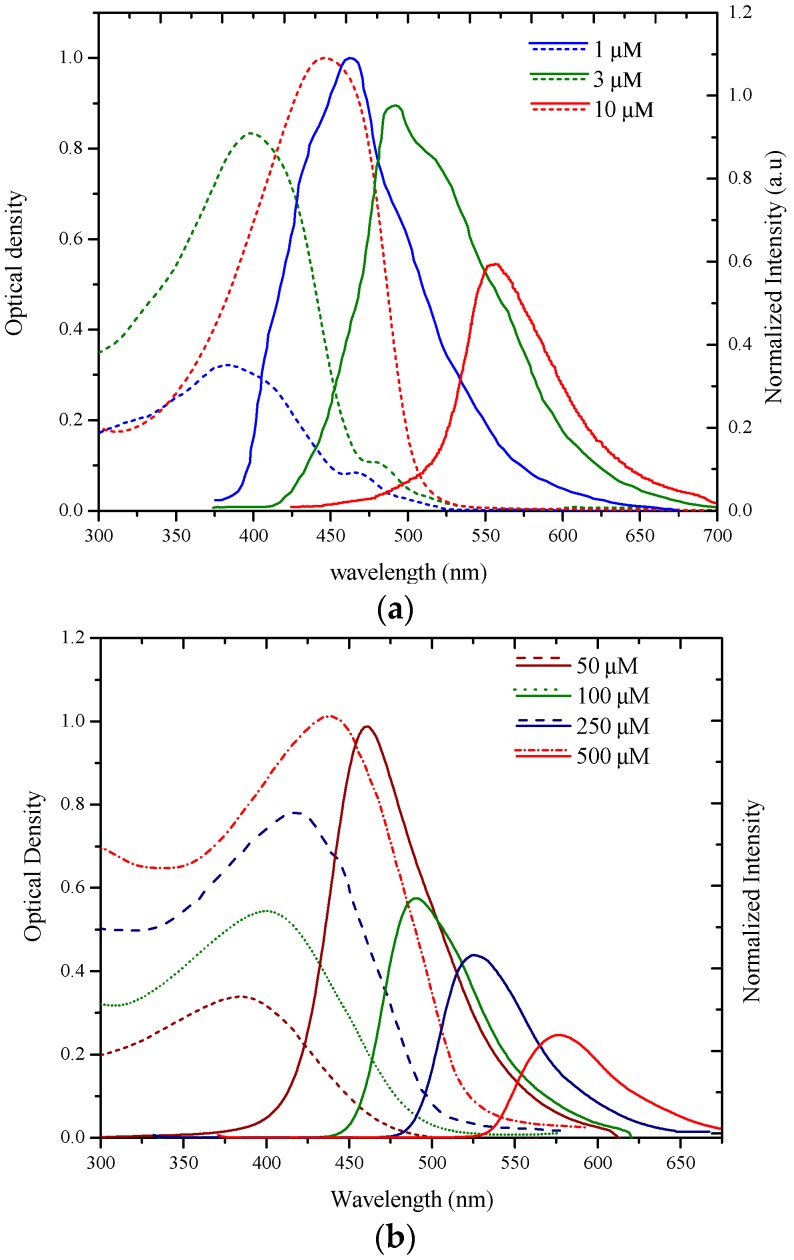
(**a**) The absorption spectra (dotted lines) and fluorescence spectra (in solid line) of the MEH–PPV in THF for three concentrations (1 to 10 µM). The γ-radiation dose was maintained at 2 kGy for each solution; (**b**) The absorption spectra and fluorescence spectra of the BMP in THF for three concentrations (50 to 500 µM). The γ-radiation dose was maintained at 2 kGy for each solution. (In the above figures, discontinuous line represents absorption and continuous line represents fluorescence).

The MEH–PPV solutions (1, 3, and 10 µM) were exposed to gamma radiation. The dose of the exposure was fixed at 2 kGy for each solution. The temperature during the radiation exposure was maintained at a constant of 5 °C. From Figure 3a, it is evident that the absorption spectra profiles had shifted to the blue region as shown (in dashed lines) [27]. However, for the BEHP-*co*-MEH–PPV, solutions of 5, 100, 250, and 500 µM were used. The absorption spectra profiles were noted to remain unchanged irrespective of the concentration, but were found to shift towards the shorter wavelength region (Figure 3a) [22].

The fluorescence spectrum of BMP in a low concentration of THF (at 1 µM) was recorded. The result revealed only a single fluorescence peak around 535 nm. As the concentration rose, the intensity declined, and the BMP fluorescence peak shifted towards the red end of the spectrum. For example, at 1 µM, the fluorescence peak was 535 nm; at 500 µM, the fluorescence peak was 580 nm. At concentrations above 500 µM, no red shift was observed, and the fluorescence peaks stayed fixed at 580 nm, as seen in Figure 2b.

Figure 3a shows the fluorescence spectra (in solid line) of the MEH–PPV in THF for three concentrations (1 to 10 µM). The γ-radiation dose was maintained at 2 kGy for each solution. At a 1 µM concentration, the spectrum profile was shifted to 455 nm (a blue shift of 105 nm from 560 nm, see Figure 2a). At concentrations of 3 and 10 µM, the blue shifts were 78 and 45 nm, respectively. At a 50 µM concentration, no significant blue shift was observed. In Figure 3b fluorescence peaks (wavelength (nm)) of BMP in THF for four different solutions (50, 100, 250, and 500 µM) can be seen. The gamma-irradiation dose was maintained at 2 kGy for each solution. At a 5 µM concentration, the spectrum profile blue shifted to 82 nm (from 542 nm corresponding to pre-radiation peak, see Figure 2b), whereas, at 100 and 250 µM, the blue shifts were 70 nm (from 560 nm) and 43 nm (from 568 nm), respectively. At a still higher concentration (500 µM), only a small blue shift (4 nm) was observed. It is noteworthy that the blue shift was less when compared with that of the MEH–PPV (ignoring the concentration difference because at these concentrations the spectral performance was comparable). These results enable the deduction that gamma-irradiation has a higher effect at lower concentrations than at high concentrations (Figure 3a,b).

**Figure 4:**
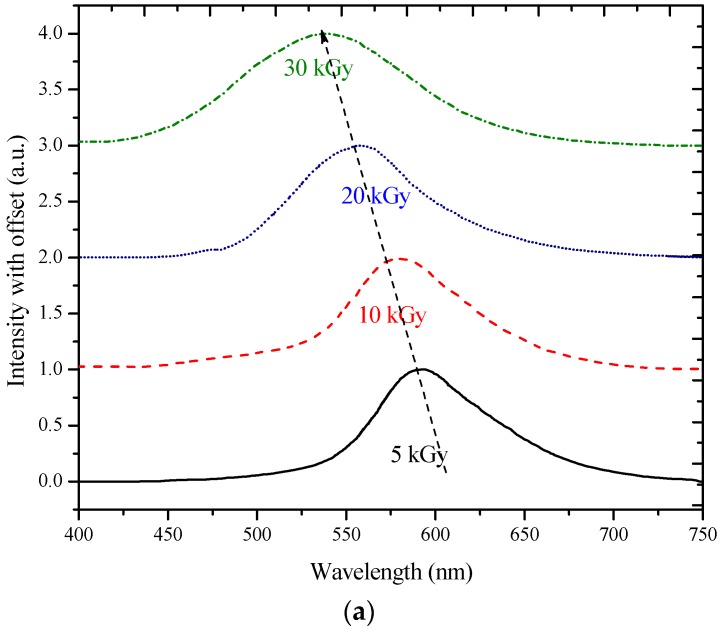

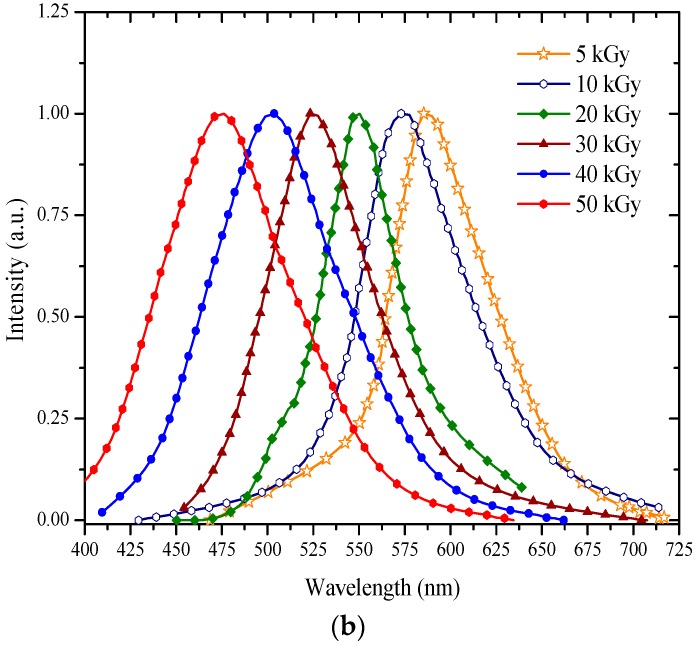
(**a**) Fluorescence spectra of MEH–PPV at 50 µM concentration, pre- and post-gamma irradiation ranging from 5 to 30 kGy; (**b**) Fluorescence spectra of BMP at 1 mM concentration, pre- and post-gamma irradiation ranging from 5 to 50 kGy.

In order to study the effect of γ-radiation at high concentrations of MEH–PPV in THF, a 50 µM solution was used. The radiation dose was then increased from 5 to 30 kGy when the γ-radiation dose given to this solution was 5 kGy. The fluorescence spectrum profile was shifted 8 nm to the blue region with a peak at 590 nm. When the dose was raised to 10 kGy, the fluorescence spectrum profile was seen to move 20 nm towards the blue region, and the peak was formed at 578 nm. At a 20 kGy dose, the fluorescence peak was around 556 nm. Finally, at a 30 kGy dose, the blue shift was at 62 nm (see Figure 4a). Similarly, for the BMP in THF, the concentration was maintained at 1 mM, while the gamma dose was varied from 5 to 50 kGy. At the 5 kGy dose the fluorescence spectrum profile shift was shown in (Figure 4b). The greatest shift was at 114 nm (refer to Table 1 for details regarding the blue shift).

### 3.2. ASE Spectra

When organic dyes such as coumarin or rhodamine are dissolved in alcohol and optically laser excited, the fluorescent dye molecules intensely absorb the incident photons and population inversion, a necessary condition for laser action, is attained. This leads to spontaneous emission and stimulated emission. If the inversion is sufficiently high, the optical gain obtained along the direction of pumping is sufficient to generate a laser-like emission called amplified spontaneous emission (ASE) or superradiant laser (SR). No feedback is needed for this mirrorless laser; single-pass gain is enough for these active media, and photons are wave guided to produce ASE. Many oligomers, conjugated polymers, and conventional laser dyes belong to this category of materials. Intrinsically, a SR is a sign of excellence laser ability of the medium, because any medium displaying a SR has the capacity to produce a laser, whereas the opposite is not true (e.g., He–Ne or Ar ion are good laser media, but do not produce ASE) [28]. In general, spectral narrowing (i.e., full width at half-maxim (FWHM)) by a factor of 2 from FWHM fluorescence, emerging as a highly directional cone of light, is proof for ASE.

The ASE spectrum of the MEH–PPV in THF at a 50 μM concentration was pumped using the third harmonic of the Nd:YAG laser (λ = 355 nm) prior to γ-radiation. The result obtained showed the ASE peak located at 600 nm with a narrow 7 nm spectral bandwidth (FWHM). This peak coincided with the maximum fluorescence emission spectrum at this concentration, as shown in Figure 2a. It is thus evident that the ASE peak has high optical gain; hence, the ASE had been produced from the excimeric state [29].

**Figure 5:**
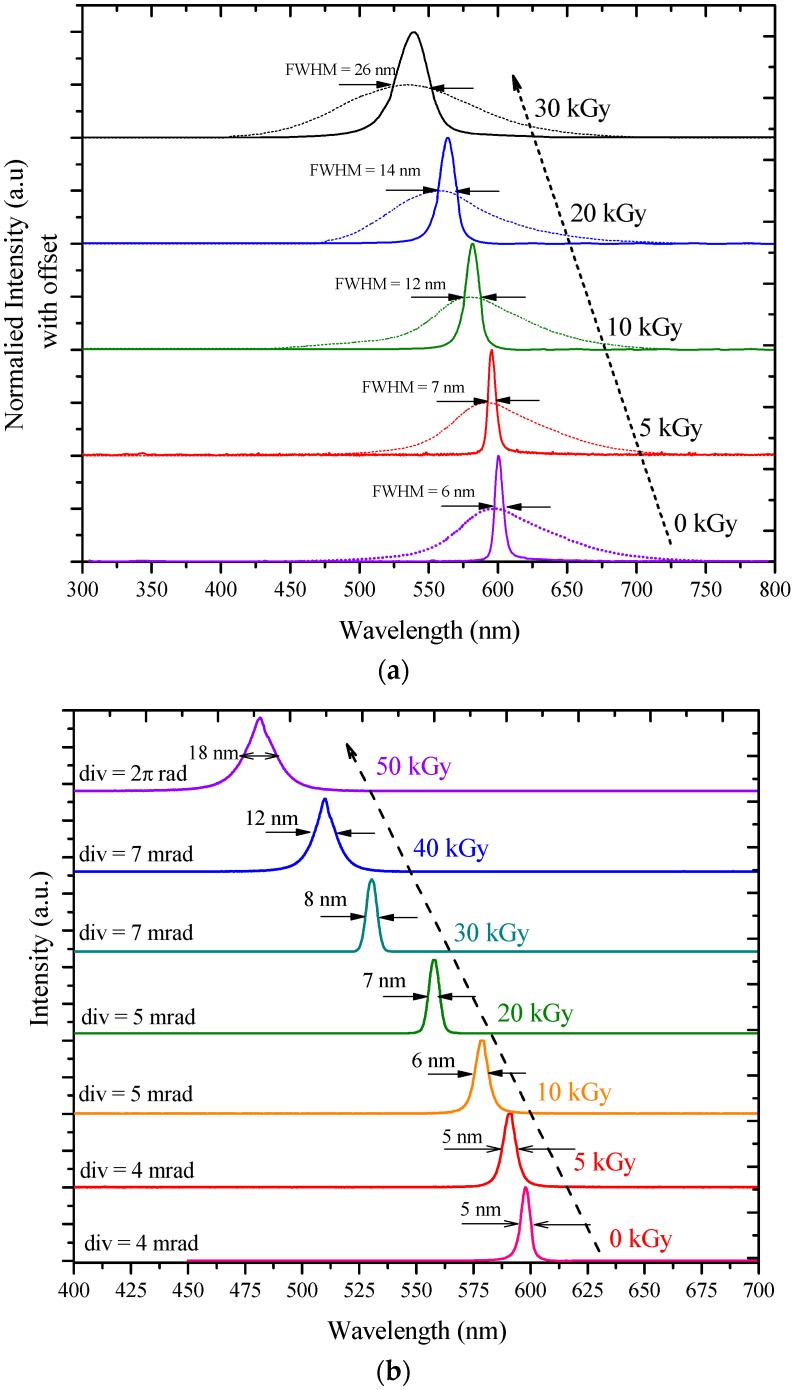
(**a**) The ASE spectrum of the MEH–PPV in THF at 50 μM concentration for different dose ranging from 5 to 30 kGy; dotted line represents laser induced fluorescence (LIF) and solid line represents Amplified Spontaneous Emission (ASE). (**b**) The ASE spectrum of the BMP in THF at 5 mM concentration for different dose ranging from 5 to 50 kGy.

The ASE spectra of MEH–PPV in THF at a 50 μM concentration under different γ-radiation doses (5 to 20 kGy) were investigated as seen in Figure 5a. The ASE spectrum was recorded under a constant pump energy of 9 mJ. At exposure to the 5 kGy dose, the ASE was found to be 594 nm. When the dose was increased (10 kGy), the ASE blue shifted at 582 nm. On further increasing the dose to 20 kGy, a blue shift of 35 nm in the ASE spectrum was observed, with a peak at 565 nm.

Both conjugated-polymers underwent spectral and spatial broadening when the irradiation dose was increased. The fluorescence and ASE spectral broadening could have been due to increased scattering contributed by crosslinking, as seen in Figure 4 and Figure 5. The crosslinking increased the polydispersity and polymer clustering, which in turn induced non-radiative losses and hence contributed to spectral broadening [30].

The alterations in the ASE properties could be ascribed to the change in the chemical structure of the conjugated (MEH–PPV) polymer. It could have occurred chiefly through the crosslinking of some bonds. The blue shift could also be due to the reduced conjugation length. For better visualization, it must be noted that all the ASE spectra were normalized and placed one over the other; moreover, the fluorescence spectra were normalized and divided by 2. The ASE amplitudes are very high in comparison to the intensity of the fluorescence, but the main objective was to depict the spectral profile and blue shift.

Earlier [22], it was demonstrated that the BMP produced ASE only in the monomeric state (560 nm) because the MEH–PPV segment of the compound was found in lower concentrations. However, at a higher concentration, between 5 and 100 mM, triple band ASEs were produced, attributed to the monomer, excimer, and double excimer of the MEH–PPV. Figure 5b shows the ASE spectra of the BMP in THF at a 5 mM concentration prior to gamma irradiation. The ASE peak was identified at 598 nm, with a narrow 5 nm spectral bandwidth (FWHM). The ASE at 598 nm was coincident with the fluorescence at this concentration.

The ASE spectra of the BMP in THF were recorded at a 5 mM concentration, after exposure to different gamma doses (5–50 kGy), as shown in Figure 5b. The pump power energy was maintained at 16 mJ. At the 5 kGy dose, the ASE was found to be 590 nm with an FWHM of 5 nm. When the dose was raised (10 kGy), the FWHM remained at 5 nm, while the ASE showed a blue shift to 579 nm. A further increase in the dose to 20 kGy induced the ASE peak to shift to 557 nm with an FWHM of 7 nm. At an even higher dose of 30 kGy, the ASE peaks shifted to 530 nm (FWHM = 8 nm) and then to 510 nm (FWHM = 12 nm) for 40 kGy. However, at 50 kGy, while laser-induced fluorescence with FWHM of 17 nm was observed, no ASE was detected. These changes occurred because of the crosslinking in these CPs. 

The spectral properties of the BMP basically originate from the MEH–PPV segment; therefore, very high concentration and pump power are required to obtain an ASE at 598 nm, by overcoming the steric hindrance of the BEHP segment [22]. In the case of the MEH–PPV in solution, the gamma radiation affects the ASE properties for doses higher than 20 kGy. However, the BMP was found to produce ASE at doses as high as even 40 kGy. This was possible perhaps because the BEHP segment protects the MEH–PPV segment and undergoes crosslinking of the polymer chains. Some preliminary experiments discussed below support this argument. However, further experiments are required to identify the chemical changes that occur, which is beyond the scope of this paper. 

**Figure 6:**
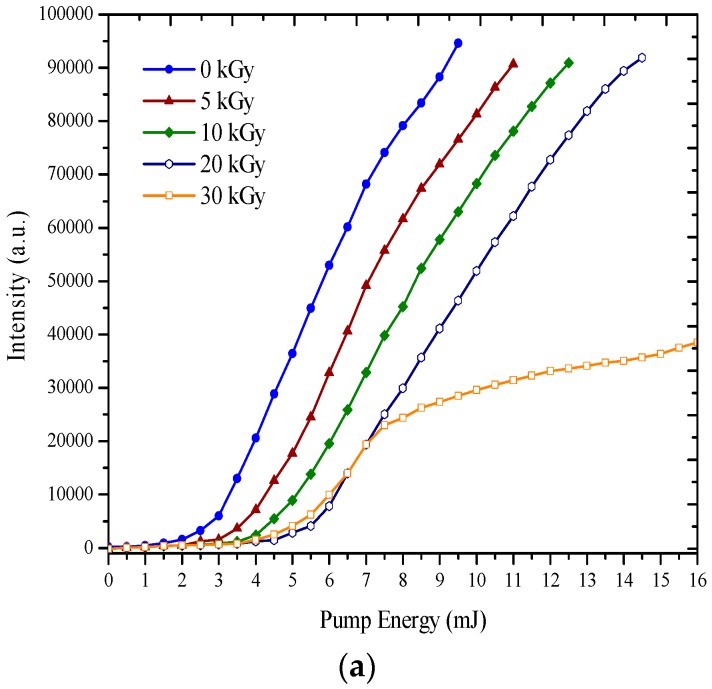

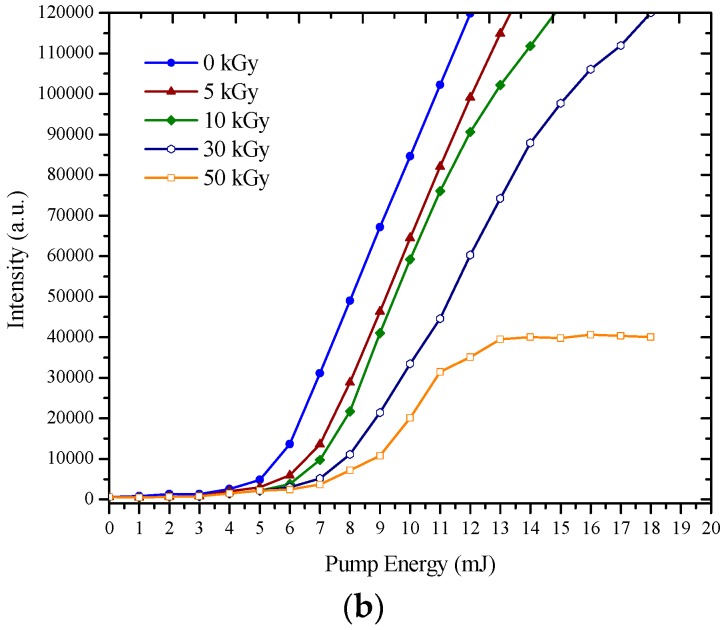
(**a**) The relationship between Pump Energy (mJ) vs. Intensity (a.u.) for MEH–PPV in THF at a 50 μM concentration for different dose ranging from 5 to 30 kGy; (**b**) The relationship between Pump Energy (mJ) vs. Intensity (a.u.) for BMP in THF at 5 mM concentration for different dose ranging from 5 to 50 kGy.

Another effect of gamma irradiation on the ASE spectra was the drop in the output intensity, shown in Table 1. The CPs in THF were prepared at a 100 µM concentration, and one part of this solution was subjected to gamma irradiation of 10 kGy. These solutions were excited by the third harmonic of an Nd:YAG laser (λ = 355 nm) under different pump energies between 6 and 12 mJ. The ASE intensity changes versus pump power energy were recorded pre- and post-exposure to the gamma irradiation. It was found that, as the pump power rose, the intensity of the CPs prior to gamma irradiation increased much faster than the CPs post gamma irradiation, as shown in Figure 6a for MEH–PPV and Figure 6b for BMP. Figure S1a,b show the actual ASE spectra, i.e., when pump energy was 8 and 11 mJ for MEH–PPV and BMP, respectively.

The wavelength tunability of the ASE spectrum by gamma irradiation could find use as a tool to obtain a broadband tunable laser from a single polymer. The amount of gamma radiation can be controlled to tune the emission wavelength to cover the different parts of the visible spectrum using a single polymer and mix them together to form a broadband tunable laser—in this case, from 450 to 610 nm; however, the drawback would be poor photochemical stability.

**Figure 7:**
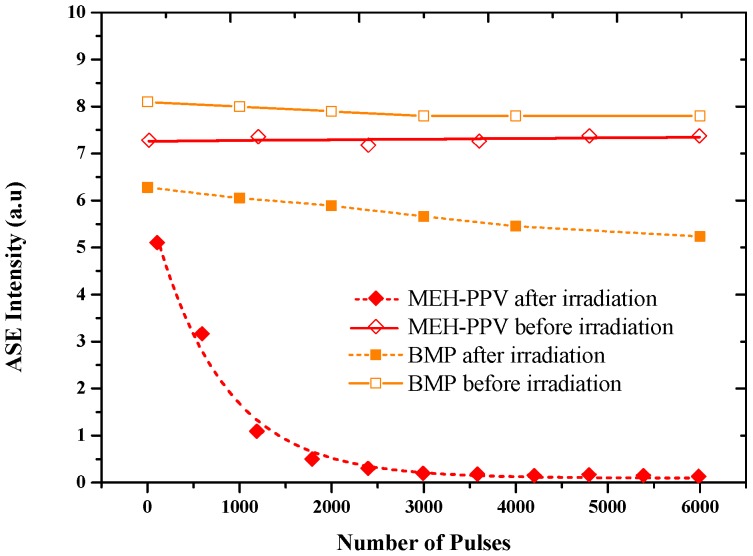
The photochemical stability of the CPs at a 100 µM concentration.

Figure 7 shows the photochemical stability of the CPs at the 100 µM concentration pre- and post-exposure (20 kGy). The solution was pumped by the third harmonic (355 nm) of the Nd:YAG laser with a pulse energy of 9 mJ and a repetition rate of 10 Hz. The ASE intensity dropped to 22% of the initial intensity after 1000 pulses and continued to appear more or less stable even after 3000 pulses; after 6000 pulses, a 33% drop was reported for the BMP. The ASE intensity dropped to 85% of the initial intensity after 1000 pulses and almost disappeared after 3000 pulses in the case of MEH–PPV.

### 3.3. Raman Spectra

**Figure 8:**
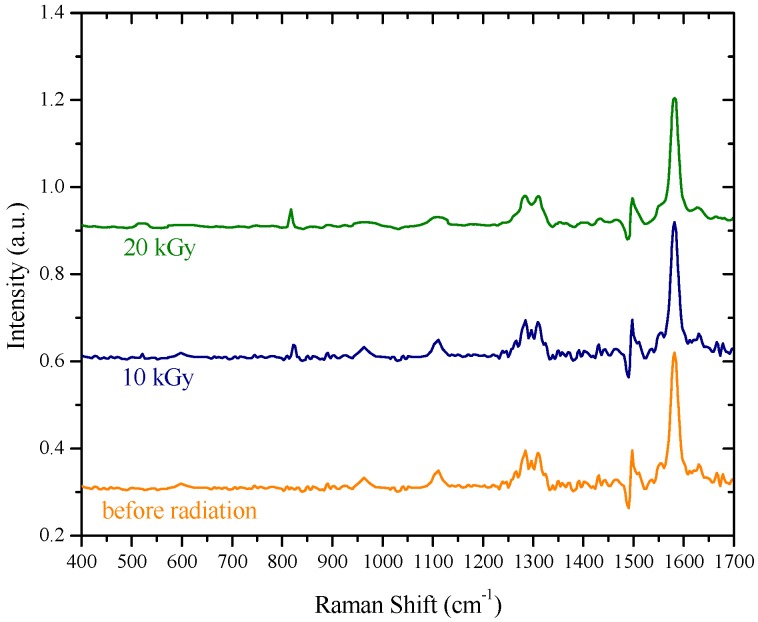
Raman spectra of the samples pre- and post-irradiation of MEH–PPV at a concentration of 20 μM.

The samples for investigation of the Raman spectra are prepared utilizing the MEH–PPV solution at a 20 μM concentration. Each sample was converted into a sheet by spin coating at a speed of 500 rpm for 20 s, and the dried samples were carefully peeled off as film. In Figure 8, the Raman spectra of the samples pre- and post-irradiation are seen, and the characteristic bands of the MEH–PPV are visible at P1 around 1553 cm^−1^, P2 around 1583 cm^−1^, and P3 around 1622 cm^−1^, which could be assigned to the C=C stretching in the vinyl group. The new peak produced at around 870 cm^−1^ indicates the formation of new small bonds, which could be attributed to crosslinking [20]. We speculate that this crosslinking could modify the chemical structure in turn increase the HOMO–LUMO gap and thus blue shift the fluorescence spectra. Additionally, the drop in the absolute peak intensity could be attributed to a reduction in the vibrational amplitude, which is due to the irradiation-induced crosslinking of the MEH–PPV. It must be noted that the peak intensities are not to scale [31,32].

### 3.4. FT-IR Spectra

**Figure 9:**
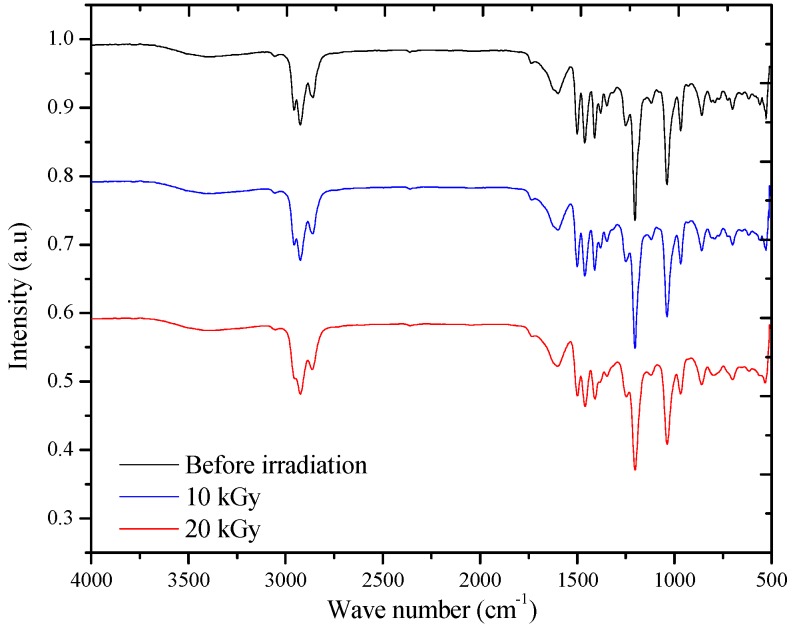
FT-IR spectra of the BEHP-*co*-MEH–PPV (BMP) samples pre- and post-irradiation at a 20 μM concentration.

Figure 9 shows the FT-IR spectra of the BEHP-*co*-MEH–PPV (BMP) samples pre- and post-irradiation at a 20 μM concentration. No significant decrease in the characteristic BMP bands or increase in the carbonyl groups (around 1650–1700 cm^−1^) was noted. The results support the hypothesis that gamma radiation does not degrade the material but instead mainly causes polymer crosslinking. To support this argument, swelling experiments, which provided direct evidence, were performed [31].

### 3.5. Swelling Experiment and Gel Permeation Chromatography (GPC)

The swelling experiment was performed for the drop-casted thin films (0, 10, and 20 kGy) made from a BEHP-co-MEH–PPV solution of 100 μM, pre- and post-radiation. The samples were dried and peeled carefully and cut into 6 mg rectangular sheets. These samples were dropped in benzene for 10 min. The excess benzene was then removed, and the swollen polymer was weighed. The weight of the fresh sample (0 kGy) and that of the acquired weight of 60 mg was recorded; for the 10 kGy sample, the weight gain was 47 mg, whereas, for the 20 kGy sample, the weight gained was 29 mg (see Table 2). This shows that, as the radiation dose increases, the crosslinking of the polymer is also increased [33].

To compare the molecular weights (*M*_w_) ratio of linear to crosslink polymer, GPC measurement was performed for both conjugated-polymers. This revealed that, prior to gamma radiation, *M*_w_ was close to the data sheet value, but the post-irradiation sample showed an increased molecular weight, which is shown in Table 2 for MEH–PPV and BMP, respectively [32].

## 4. Conclusions

In this study, the spectral and ASE properties of the conjugated polymers in THF at different concentrations were obtained, pre- and post-gamma irradiation, using different gamma radiation doses. The effects of gamma irradiation on the fluorescence and ASE of the CPs at different doses and concentrations have been demonstrated. The ASE spectra of the CPs after gamma irradiation were observed to be shifted to the blue end of the spectrum as the dose increased. The results also highlighted that the BEHP-*co*-MEH–PPV had stronger photochemical stability pre- and post-gamma irradiation compared with the MEH–PPV.

## Figures and Tables

**Table 1:** (**a**) The details of BMP fluorescence, ASE peak wavelength (nm), peak intensity (a.u.), and blue shift occurring due to gamma radiation and full width half maximum (FWHM) of ASE when pump energy was 11 mJ; (**b**) The details of MEH–PPV fluorescence, ASE peak wavelength (nm), peak intensity (a.u), and blue shift occurring due to gamma radiation and full width half maximum (FWHM) of ASE when pump energy was 8 mJ.

	BEHP-*co*-MEH–PPV					
	Dose kGy	Fluorescence peak	ASE peak wavelength (nm)	Peak intensity (a.u.)	Blue shift	FWHM
**a**	0	595	598	102,045	0	5
	5	585	590	81,081	5	5
	10	573	579	76,091	19	7
	20	550	557	70,832	41	7
	30	525	530	44,390	68	8
	40	504	510	36,067	88	12
	50	476	481	31,434	117	17
	**MEH–PPV**					
**b**	0	595	600	78,985	0	6
	5	590	595	61,224	5	7
	10	578	582	45,012	18	12
	20	556	563	29,587	37	14
	30	536	540	23,971	60	28

**Table 2:** (**a**) Quantum yield, molecular weight and polydispersity of MEH–PPV; (**b**) Quantum yield, molecular weight and polydispersity of BMP.

	MEH–PPV			
	Dose kGy	Quantum yield	Molecular weight (*M*_w_)	Polydispersity index PDI = $\frac{M_{w}}{M_{n}}$
**a**	0 kGy	0.43	120,830	1.6
	10 kGy	0.32	144,508	2.5
	20 kGy	0.27	159,204	3.6
**BMP**				
**b**	0 kGy	0.46	132,030	2.4
	10 kGy	0.35	142,310	3.2
	20 kGy	0.28	156,025	4.1

## Supplementary Materials

The following are available online at www.mdpi.com/2073-4360/9/1/7/s1, Figure S1: (a) ASE intensity (a.u.) of post-irradiation sample of MEH–PPV at a concentration of 50 μM and a pump energy of 8 mJ; (b) ASE intensity (a.u.) of post-irradiation sample of MEH-PPV at a concentration of 5 mM and a pump energy of 11 mJ.
