# Supplementary material for: Gamma-Irradiation Effects on the Spectral and Amplified Spontaneous Emission (ASE) Properties of Conjugated Polymers in Solution
Source: Polymers (Basel). 2016 Dec 28;9(1):7. doi: 10.3390/polym9010007 (PMC6432246; doi:10.3390/polym9010007)
Supplement: Supplementary file 1 [file polymers-09-00007-s001.pdf]

Supplementary Materials: Gamma-Irradiation Effects on the Spectral and Amplified Spontaneous Emission (ASE) Properties of Conjugated Polymers in Solution

Mohamad S. AlSalhi, Saradh Prasad, D. Devaraj and Ziad S. Abo Mustafa

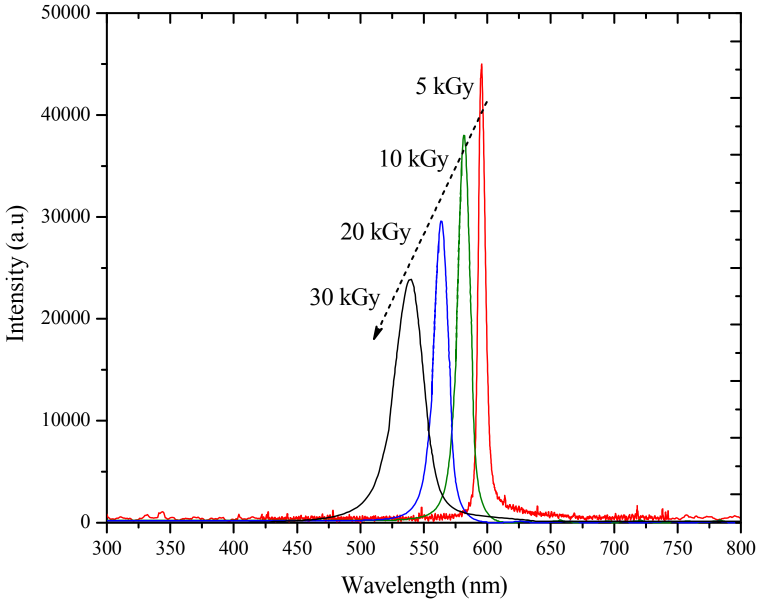

(**a**)

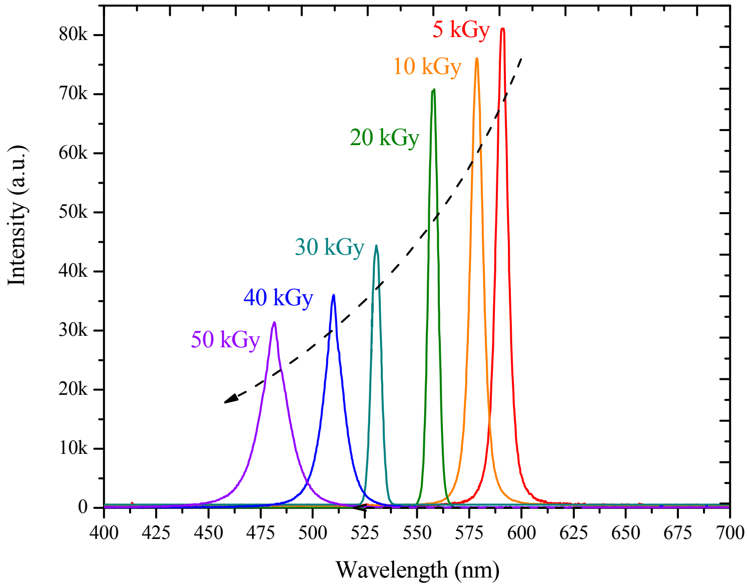

(**b**)

Figure S1. (**a**) ASE intensity (a.u.) of post-irradiation sample of MEH–PPV at a concentration of 50 μM and a pump energy of 8 mJ; (**b**) ASE intensity (a.u.) of post-irradiation sample of MEH-PPV at a concentration of 5 mM and a pump energy of 11 mJ.
